# Exploring the Feasibility of Deep Learning for Predicting Lignin GC-MS Analysis Results Using TGA and FT-IR

**DOI:** 10.3390/polym17060806

**Published:** 2025-03-18

**Authors:** Mingyu Park, Byung Hwan Um, Seung-Hyun Park, Dae-Yeol Kim

**Affiliations:** 1 Department of Computer Engineering, Kyungnam University, Changwon 51767, Gyeongsangnam-do, Republic of Korea; parkmingyu711@gmail.com; 2 Carbon-Neutral Resources Research Center, Hankyong National University, 327, Jungang-ro, Anseong 17579, Gyeonggi-do, Republic of Korea; bhum11@hknu.ac.kr; 3 School of Food Biotechnology and Chemical Engineering, Hankyong National University, 327, Jungang-ro, Anseong 17579, Gyeonggi-do, Republic of Korea; 4 Fivenode, Seoul 07549, Republic of Korea; ppillip@gmail.com; 5 Department of Artificial Intelligence, Kyungnam University, Changwon 51767, Gyeongsangnam-do, Republic of Korea

**Keywords:** depolymerized lignin prediction, deep learning, GC-MS analysis, biomass valorization, multimodal spectroscopic analysis

## Abstract

Lignin is a complex biopolymer extracted from plant cell walls, playing a crucial role in structural integrity. As the second most abundant biopolymer after cellulose, lignin has significant industrial value in bioenergy, the chemical industry, and agriculture, gaining attention as a sustainable alternative to fossil fuels. Its composition changes during degradation, affecting its applications, making accurate analysis essential. Common lignin analysis methods include Thermogravimetric Analysis (TGA), Fourier-transform Infrared Spectroscopy (FT-IR), and Gas Chromatography–Mass Spectrometry (GC-MS). While GC-MS enables precise chemical identification, its high cost and time requirements limit frequent use in budget-constrained studies. To address this challenge, this study explores the feasibility of an artificial intelligence model that predicts the GC-MS analysis results of depolymerized lignin using data obtained from TGA and FT-IR analyses. The proposed model demonstrates potential but requires further validation across various lignin substrates for generalizability. Additionally, collaboration with organic chemists is essential to assess its practical applicability in real-world lignin and biomass research.

## 1. Introduction

Lignin is a major component of the plant cell wall, along with cellulose and hemicellulose. Traditionally considered a by-product of cellulose production, it has often been discarded or used as fuel. However, with increasing emphasis on sustainability, lignin is gaining attention as a potential fossil fuel alternative across various industries and remains an active research focus in the chemical industry, bioenergy, and agriculture [1,2,3].

Composed of monomeric units such as p-hydroxyphenyl, guaiacyl, and syringyl, lignin forms a complex three-dimensional structure stabilized by various linkages (β-O-4, β-β, and 5-5) and functional groups, including methoxy, carboxyl, and carbonyl moieties [4]. The heterogeneous nature of lignin complicates its utilization as a uniform chemical feedstock in industrial applications, particularly in bio-based material synthesis and biorefinery processes [5].

To enhance lignin utilization, depolymerization is essential. Methods include thermal decomposition, chemical treatments with oxidizing or reducing agents, and catalytic processes. Among these, hydrothermal liquefaction (HTL) is widely applied, converting high-molecular-weight biomass into low-molecular-weight aromatic compounds, such as bio-oil, gas, and solid residues [6].

Since the physicochemical properties of depolymerized lignin are strongly influenced by the processing method, precise compositional analysis is essential for optimizing downstream applications such as bio-oil production and polymer synthesis [7,8]. Common techniques include Thermogravimetric Analysis (TGA), Fourier-transform Infrared Spectroscopy (FT-IR), and Gas Chromatography–Mass Spectrometry (GC-MS). This study utilizes these methods to analyze depolymerized lignin.

TGA assesses thermal stability by monitoring decomposition under controlled heating, while FT-IR identifies functional groups based on their infrared absorption profiles. GC-MS delivers comprehensive qualitative and quantitative analysis of complex mixtures and remains the method of choice in many research settings [9,10,11,12,13].

However, the high capital and operational costs of GC-MS instruments (typically USD 50,000–USD 150,000) often limit in-house analyses, forcing researchers to outsource sample testing at significant per-sample costs (USD 100–USD 500, compared to USD 50–USD 150 for TGA and USD 20–USD 100 for FT-IR [14]), thereby restricting the scale and frequency of GC-MS applications in resource-limited studies. These economic constraints pose significant challenges, particularly in studies involving extensive sample analysis.

Recent advancements in artificial intelligence (AI) have facilitated more efficient lignin analysis, particularly in response to financial and logistical constraints associated with conventional chemical techniques. Wen et al. (2024) developed a predictive model leveraging FT-IR and machine learning (XGBoost, LightGBM) to estimate lignin content, demonstrating that this approach significantly accelerates analysis compared to traditional methods [15]. Ge et al. (2023) employed machine learning to model lignin structural transformations during DES pretreatment, confirming that delignification dynamics and structural modifications can be reliably predicted with minimal experimental data [16]. Diment et al. (2024) utilized Bayesian optimization to enhance the production of lignin–carbohydrate complexes (LCCs), demonstrating that machine learning can optimize biorefinery conditions, reducing the need for extensive experimental trials while improving yield and structural control [17]. Löfgren et al. (2022) applied Bayesian optimization to infer lignin properties without direct experimentation, facilitating the derivation of optimal compositions [18].

Existing studies utilizing machine learning for lignin characterization predominantly focus on macroscopic property estimation, such as structural changes and functional group analysis. In contrast, this study specifically aims to predict the individual chemical composition of lignin depolymerization products by leveraging deep learning models trained on TGA and FT-IR data. To address this limitation, this study introduces a deep learning-based framework that utilizes cost-effective TGA and FT-IR data to predict GC-MS analytical results. The key contributions of this research are as follows:

1. **Decoder-Based Generative Models**: A deep learning model incorporating the distinct features of TGA, FT-IR, and GC-MS data to generate synthetic datasets.
2. **Predictive Modeling in Limited Data Scenarios**: Synthetic data augmentation to enable training under data-scarce conditions.
3. **GC-MS Predictability Using TGA and FT-IR Data**: Development and evaluation of a GC-MS prediction model based on TGA and FT-IR inputs.

This paper is organized as follows: Section 2 provides details on dataset preprocessing methods and the generative dataset model. Section 3 introduces the proposed predictive model. Section 4 analyzes the results, highlights key findings, and suggests future research directions.

## 2. Proposed Method

This section describes the process of developing a deep learning-based GC-MS prediction model using data preprocessing methods and data generation deep learning models.

**Figure 1:**
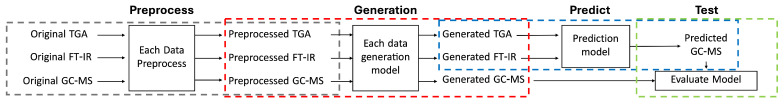
Overall research workflow.

Figure 1 illustrates the overall research workflow. Due to the limitations of the available data, we employed a data generation strategy that encapsulates the inherent characteristics of the data, facilitating the generation of supplementary samples. This study investigates the potential of predicting GC-MS data using TGA and FT-IR datasets.

Recent research highlights the prevalence of Convolutional Neural Network (CNN)-based data generation and augmentation methodologies across various domains. These approaches effectively mitigate the constraints posed by limited experimental data and enhance the efficacy of predictive models [19,20,21]. Consequently, we implemented CNN-based data augmentation techniques to expand both TGA and FT-IR datasets. The augmented TGA and FT-IR datasets are then utilized as inputs for the GC-MS prediction model, which aims to estimate the corresponding GC-MS data. The predicted GC-MS results are subsequently compared with the augmented GC-MS data.

Through this approach, this study examines the feasibility of the deep learning-based GC-MS prediction model.

### 2.1. Dataset Overview

The datasets analyzed in this study were collected in a laboratory environment. The provided data include analysis results from three methods: TGA, FT-IR, and GC-MS. These methods were used to study depolymerized lignin heated at four different temperatures (250 °C, 300 °C, 350 °C and 400 °C) during the HTL process. Each method contains experimental results for all four temperatures.

TGA analysis was performed on depolymerized lignin under HTL temperature conditions of 250 °C, 300 °C, 350 °C and 400 °C. The initial temperatures of the TGA data were 39.65 °C, 40.42 °C, 39.62 °C, and 39.61 °C, while the final temperatures were 787.58 °C, 785.94 °C, 787.52 °C, and 786.58 °C. The dataset contains approximately 22,000 data points.

FT-IR analysis was performed three times for each temperature, resulting in a total of 12 datasets. The recorded wavelength range of the data is from 649.8934 cm^−1^ to 4000.6047 cm^−1^. Characteristic chemical bonds, such as C=O at 1730 cm^−1^ and O-H at 3200–3600 cm^−1^, are identified in the spectrum, where peaks can be observed in the graph.

The compounds detected through GC-MS analysis are classified into nine groups, each comprising multiple derivatives, totaling 114 chemical compounds. The analysis results indicate the relative proportions of depolymerized lignin components represented by each derivative. Detailed information on the identified compounds is provided in Appendix A.

#### 2.1.1. Tga Data Preprocessing and Generation

**Figure 2:**
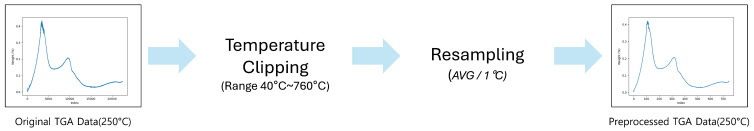
TGA data preprocessing pipeline.

Figure 2 illustrates the proposed TGA data preprocessing pipeline, which consists of two stages. Initially, temperature clipping was conducted. The depolymerized lignin was analyzed via TGA at HTL temperatures of 250 °C, 300 °C, 350 °C, and 400 °C. The initial temperatures of the TGA data recorded were 39.65 °C, 40.42 °C, 39.62 °C, and 39.61 °C, with final temperatures measuring 787.58 °C, 785.94 °C, 787.52 °C, and 786.58 °C. However, a notable discrepancy in the initial and final temperatures was observed. This variance can be attributed to factors such as the operational conditions of the TGA apparatus, the initial status of the sample, and the automatic shutdown parameters. Beyond 700 °C, the stabilization of residual carbon and the thermal changes progressively decrease. In particular, data collected above 760 °C may be influenced by noise arising from the thermal constraints of the TGA instrument at elevated temperatures, thereby compromising data reliability. To mitigate this concern, the data range was confined to an initial temperature of 40 °C and a final temperature of 760 °C, ensuring consistency in the temperature gradients.

Furthermore, a resampling process was applied. The dataset comprised over 22,000 data points, which, if used for training, could lead to overfitting and require long training durations. To address this challenge, the weight change data were averaged at 1 °C intervals for each temperature, thereby reducing the dataset size. This methodological approach facilitated the elimination of redundancy and minimized the risk of overfitting [22].

Table 1 shows the proposed TGA data generation model architecture. The TGA data are a one-dimensional time series dataset that records the weight of a sample as the temperature increases. This characteristic was considered in the model design, leading to the adoption of the CNN architecture. To enable the model to learn the causal relationship between weight changes and temperature variations, two sets of Conv+ReLU layers were placed to extract local patterns. Subsequently, a Fully Connected layer (FC) integrates global features to generate the final TGA data. This structure captures the sequential nature of TGA data, and its decoder-based design effectively reflects TGA characteristics, allowing data generation under various conditions.

#### 2.1.2. FT-IR Data Preprocessing and Generation

**Figure 3:**
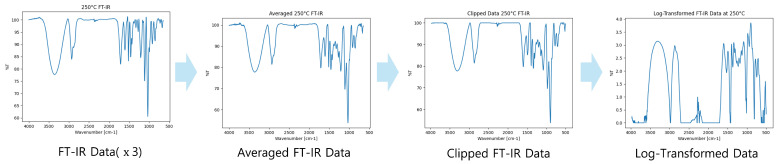
FT-IR data preprocessing pipeline.

Figure 3 illustrates the FT-IR data preprocessing pipeline. An analysis of the provided FT-IR datasets revealed a slight exceedance of the 100% threshold due to noise and outliers. Since FT-IR data are used to assess the spectral absorbance or transmittance of samples, values exceeding 100% pose inherent issues, necessitating a preprocessing step to prevent the generation of extraneous or distorted data during model training. The dataset comprises results from three analyses conducted under uniform conditions, resulting in a total of 12 distinct datasets. Figure 3 illustrates the process of integrating these datasets, addressing the aforementioned issues, and transforming them into a format optimized for efficient model training. This integration can be mathematically articulated as follows.

(1) $$D_{\mathrm{preprocessed}}=\log\left(\max\left(101-\min\left(\frac{1}{N}\sum_{i\in \mathrm{Group}}D_{\mathrm{FTIR},i},100\right),10^{-6}\right)\right)$$

In Equation (1), $D_{\mathrm{FTIR},i}$ represents the FT-IR spectrum data of the *i*-th sample, and preprocessing is carried out through the following procedure.

The data are first integrated by calculating the arithmetic mean for each temperature. Subsequently, values exceeding 100 are constrained using the minimum function to ensure they remain within this threshold. The clipped mean values are then subtracted from 101 to adjust for offset and stabilization.

To prevent errors during logarithmic transformation due to excessively small values, a stabilization constant of $10^{-6}$ is applied using the maximum function. Finally, a logarithmic transformation is performed to reduce the dynamic range of the data, making it more suitable for analysis.

This preprocessing enhances the reliability of FT-IR data by minimizing the influence of extreme values while improving its usability for subsequent modeling and analysis.

Table 2 shows the proposed FT-IR data generation model architecture. FT-IR data encapsulate the absorption of light by a sample across various frequency ranges, represented in the form of a spectrum. Given that this methodology yields pronounced peaks in specific frequency bands alongside inherent noise, a dropout technique has been integrated into CNN to address these nuances. Dropout is instrumental in mitigating overfitting by randomly deactivating a subset of neurons during the training process. Initially, three convolutional layers with ReLU activation extract features from distinct frequency regions, while the FC aggregates the overall spectral distribution. Subsequently, the dropout layer diminishes the propensity of the model to become biased toward noise or dominant peak values in particular frequency bands during training. This framework is conceived as a decoder-based model that faithfully represents the spectral characteristics intrinsic to FT-IR data, thereby ensuring robust data generation, even in the presence of complex FT-IR spectra.

#### 2.1.3. GC-MS Data Preprocessing and Generation

GC-MS analysis is a technique that integrates Gas Chromatography (GC) and Mass Spectrometry (MS). GC serves as the initial step, facilitating the separation of the components within a complex mixture. The sample is heated to vaporize the constituents, which are then introduced into a chromatographic column. As the gaseous components traverse the column, they interact variably with the stationary phase, resulting in differential retention times that enable the respective components to be distinguished. Following this separation, Mass Spectrometry is employed to further analyze the isolated components. The samples are directed into a mass spectrometer, where they undergo ionization. The resulting ionized molecules are subsequently separated according to their mass-to-charge ratio (m/z), with their velocities varying in correlation to their mass. This intricate process culminates in the generation of a mass spectrum, which provides critical insights into the molecular weight, structural characteristics, and elemental composition of each identified component.

During the analysis of the acquired GC-MS data, certain components were not fully quantified. For instance, several compounds within the Syringyl group were not detected in the dataset utilized for this study. These compounds include 4-Propylsyringol (2,6-Dimethoxy-4-propylphenol), Propiosyringone (1-Propanone, 1-(4-hydroxy-3,5-dimethoxyphenyl)-), and Dihydrosyringenin (3-Syringylpropanol). Consequently, the total proportion of detected components at each temperature condition (250 °C, 300 °C, 350 °C, and 400 °C) was 91.16%, 91.23%, 86.11%, and 88.07%, respectively, falling short of the expected sum of 100%.

This phenomenon can be attributed to multiple factors, including insufficient volatility that hinders the migration of the compound through the GC column, leading to its residual presence, thermal degradation at elevated temperatures which results in the loss of its original signal, and inadequate ionization within the MS process, culminating in the absence of detectable signals.

To resolve this issue, data preprocessing is performed. Initially, instead of using the proportion of each derivative within a group, the overall group proportion was considered. This methodology preserves critical information essential for model training while enhancing computational efficiency. Furthermore, an “Other” classification was introduced to account for undetectable compounds within the GC-MS analysis, thereby ensuring that the total proportion sums to 100%. This modification prevents distortions arising from incomplete data and enhances the accuracy and generalizability of the resultant findings.

Table 3 shows the proposed GC-MS data generation model architecture. This model is designed as a decoder-based CNN architecture, generating new GC-MS data using the provided input. First, the Conv+ReLU layers learn the local correlations between component ratios. Then, the Global Average Pooling (GAP) layer compresses the feature maps extracted from multiple convolutional layers into a single average value [23]. The GAP layer reduces the number of parameters, prevents overfitting, and efficiently represents the global characteristics of the input data. The global features obtained from the GAP layer are then passed to the FC to learn the overall correlations between the input component ratios and GC-MS data. Finally, the output layer, which includes a Softmax activation function, converts the probabilistic distribution of the GC-MS data into component values.

### 2.2. Proposed GC-MS Prediction Model

This section proposes a model that predicts GC-MS data using generated data from the model described in Section 2.1 as input data.

#### 2.2.1. Model Architecture

In this study, a model integrating a Multimodal Variational Auto Encoder (MVAE) [24] with a Mixture of Experts (MoE) [25] to predict GC-MS data is presented. The model encompasses an encoder that assimilates TGA and FT-IR spectroscopic data as inputs, a decoder that reconstructs data within the latent space, an expert network that forecasts GC-MS data, and a gating network that determines the weighting of the expert network.

**Figure 4:**
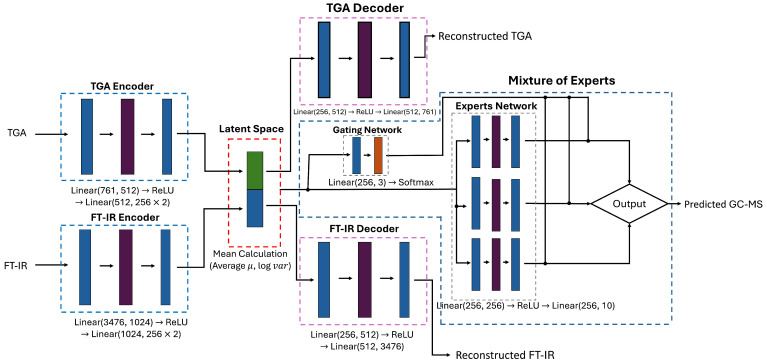
The proposed GC-MS data prediction model architecture.

Figure 4 presents the proposed GC-MS data prediction model architecture, which integrates encoders, latent space representation, decoders, and expert-based prediction using an MoE.

In the encoders, 761-dimensional TGA data and 3476-dimensional FT-IR data are processed through separate encoders. Each encoder extracts meaningful features from its respective input data, computing the mean (μ) and logarithmic variance (logσ) to define a probabilistic distribution. These encoded features are then integrated into a shared latent space, where the intrinsic characteristics of TGA and FT-IR data are fused into a unified latent representation *z*. The latent vector is sampled using the reparameterization trick, which enables the model to maintain probabilistic properties while ensuring efficient training.

The latent vector *z* serves as a central representation that facilitates both data reconstruction and predictive modeling. Two decoders independently reconstruct the original spectral data from *z*, with the TGA decoder and FT-IR decoder minimizing the reconstruction loss of the Variational Auto Encoder (VAE) [26]. Concurrently, the same latent vector is utilized for GC-MS prediction through the MoE framework.

Within the MoE structure, multiple expert networks analyze the latent vector from different perspectives to generate GC-MS predictions. A gating network dynamically assigns optimal contributions to each expert, ensuring adaptive weighting based on input characteristics. By leveraging these mechanisms, the MVAE-MoE model effectively integrates TGA and FT-IR data to improve the accuracy and generalizability of GC-MS predictions while enabling robust estimations under varying temperature conditions in the HTL process.

#### 2.2.2. Loss Function

To optimize the model training, the loss function was newly defined as follows.

(2) $$L_{\mathrm{Total}}=L_{\mathrm{Recon}}+L_{\mathrm{Pred}}+L_{\mathrm{KL}},\;\mathrm{where}\;L_{\mathrm{Recon}}=L_{\mathrm{Recon}}^{\mathrm{TGA}}+L_{\mathrm{Recon}}^{\mathrm{FT}-\mathrm{IR}}$$

In Equation (2), $L_{\mathrm{Total}}$ signifies the comprehensive loss value. $L_{\mathrm{Recon}}^{\mathrm{TGA}}$ and $L_{\mathrm{Recon}}^{\mathrm{FT}-\mathrm{IR}}$ denote the reconstruction losses associated with the TGA and FT-IR datasets, respectively, which are restored through the TGA decoder and FT-IR decoder within the model architecture.

In a VAE, the input data are encoded into a latent distribution, and the reparameterized latent vector *z* is subsequently reconstructed to closely approximate the original input. This procedure assesses the fidelity with which the latent space represents the input data. Furthermore, $L_{\mathrm{Pred}}$ quantifies the difference between the predicted GC-MS data and the actual GC-MS data, using the Mean Squared Error (MSE) as the loss function. The reconstruction and prediction loss is quantified using MSE, which can be formulated as follows:

(3) $$\left\{L_{\mathrm{Recon}}^{\mathrm{TGA}},L_{\mathrm{Recon}}^{\mathrm{FT}-\mathrm{IR}},L_{\mathrm{Pred}}\right\}=\frac{1}{n}\sum_{i=1}^{n}{\left(y_{i}-{\hat{y}}_{i}\right)}^{2}$$

In Equation (3), $y_{i}$ represents the actual data, while ${\hat{y}}_{i}$ denotes the reconstructed or predicted data. The variable *n* indicates the data index. The MSE is computed as the squared difference between the actual and predicted values, imposing greater penalties on larger errors. This mechanism guides the latent space to effectively learn the intrinsic characteristics of TGA and FT-IR data, thereby enhancing the model’s representation capability.

(4) $$L_{\mathrm{KL}}=-\frac{1}{2}\sum \left(1+\log\left(\sigma^{2}\right)-\mu^{2}-\sigma^{2}\right)$$

Equation (4) defines $K_{\mathrm{KL}}$ as the KL divergence loss function in Equation (2), which quantifies how closely the latent vector *z* approximates the standard normal distribution N(0, 1) [27]. KL divergence loss plays a crucial role in regulating the structure of the latent space by encouraging the mean (μ) to be close to 0 and the variance ($\sigma^{2}$) to be close to 1. Ensuring that μ≈0 prevents the latent variables from shifting excessively in one direction, maintaining a balanced and well-structured distribution. Similarly, enforcing $\sigma^{2}\approx 1$ prevents the latent space from collapsing into a single point or becoming overly dispersed, both of which can degrade the quality of learned representations. If $\sigma^{2}$ is too small, the latent space becomes too concentrated, limiting the diversity of generated outputs, while if it is too large, important structural information may be lost. By maintaining these constraints, the model ensures that the latent variables remain consistent with the standard normal distribution, allowing for stable training and generalization. Ultimately, KL divergence loss functions as a mechanism that prevents the latent space from becoming unstructured, thereby improving the model’s ability to generate meaningful and diverse representations.

## 3. Experimental Result

This section performs a comprehensive evaluation of each data generation model and the GC-MS prediction model introduced in the preceding section. The model training settings are as follows: the Adam optimizer is utilized with an initial learning rate of 0.001. To adjust the learning rate dynamically, the StepLR scheduler reduces it by a factor of 0.1 after 75 epochs. The batch size is set to 32, and training is conducted for 100 epochs. During training, the loss value is calculated at each epoch, followed by backpropagation to minimize the loss. In addition, loss is monitored at each epoch to track training progress.

### 3.1. Evaluate Each Data Generation Model

The evaluation methodology involves generating results across different temperature settings for each model, which are subsequently analyzed to assess model performance. However, the target temperatures that the models are intended to predict are not included in the provided dataset. Consequently, actual data are not available for direct comparison with the model predictions.

To address this limitation, interpolation was applied based on the relationships between existing data to generate a comparative dataset. Recently, data augmentation techniques based on interpolation have been utilized in various studies. This approach has led to studies aimed at overcoming the limitations of the dataset [28,29,30,31,32].

Given the limited availability of labeled experimental data, interpolation provides a viable approach to synthetically enhance data diversity while preserving key statistical properties, thereby improving the robustness of predictive models. In this study, linear interpolation was applied for comparative analysis, as defined below:

(5) $$y=y_{1}+\frac{x-x_{1}}{x_{2}-x_{1}}\left(y_{2}-y_{1}\right)$$

In Equation (5), $x_{1}$ and $x_{2}$ denote the specified temperature conditions, while $y_{1}$ and $y_{2}$ represent the GC-MS data corresponding to these temperature conditions.

Linear interpolation is an effective method when data exhibit linear characteristics; however, if nonlinear patterns are present, the interpolated values may not accurately reflect the underlying data properties. Nonetheless, this study adopted linear interpolation for its simplicity and ease of application with a limited number of data samples.

The model evaluation was performed using metrics such as the Mean Absolute Error (MAE), $R^{2}$, and the Pearson correlation coefficient. Botchkarev elucidated that distinct evaluation metrics possess unique characteristics, and reliance on a solitary metric may only capture specific dimensions of model performance [33]. Therefore, by concurrently analyzing MAE, $R^{2}$, and the correlation coefficient, we attain a more nuanced assessment of the efficacy and reliability of the model.

(6) $$\mathrm{MAE}=\frac{1}{n}\sum_{i=1}^{n}\left|y_{i}-{\hat{y}}_{i}\right|$$

Equation (6) defines the MAE [34]. The MAE quantifies the average absolute deviation between the predicted values and the interpolated values, indicating the extent to which the model predictions align with the reference values. A lower MAE value, approaching zero, signifies a stronger correspondence between the predicted and reference values.

(7) $$R^{2}=1-\frac{\sum_{i=1}^{n}{\left(y_{i}-{\hat{y}}_{i}\right)}^{2}}{\sum_{i=1}^{n}{\left(y_{i}-\bar{y}\right)}^{2}}$$

Equation (7) defines the $R^{2}$ [35], which quantifies how well the model captures the variability in the interpolated values. A value closer to 1 indicates that the model accurately reflects the changes in the interpolated values. In some cases, the $R^{2}$ value may be negative, implying that the model’s predictions perform worse than the interpolated values.

(8) $$r=\frac{\mathrm{Cov}\left(y,\hat{y}\right)}{\sigma_{y}\cdot \sigma_{\hat{y}}}$$

Equation (8) defines the correlation coefficient [36], a statistical measure that quantifies the linear relationship between predicted and actual values. This coefficient evaluates the degree to which the two datasets co-vary, with values approaching 1 indicating a strong positive linear correlation, while values near 0 suggest the absence of a discernible linear relationship.

#### 3.1.1. TGA Data Generation Model

To quantify the data loss introduced during the TGA data preprocessing, the Wasserstein distance is utilized.

(9) $$W_{1}\left(P,Q\right)=\underset{\gamma \in \Pi (P,Q)}{\inf}\int_{R^{d}\times R^{d}}\left|x-y\right|d\gamma \left(x,y\right)$$

Equation (9) defines the Wasserstein distance, which is a metric that measures the optimal transport cost between two probability distributions [37] and is used to quantitatively evaluate the differences between data distributions. In this study, the Wasserstein distance was utilized to quantify the information loss between data before and after preprocessing.

Table 4 presents the Wasserstein distance between preprocessed and original data. In Table 4, it can be observed that information loss during TGA data preprocessing was minimal. This indicates that the preprocessing method effectively preserved the intrinsic characteristics of the data while also reducing its size. Furthermore, the results suggest that the preprocessed data maintain a high degree of similarity to the original data, supporting their reliability as input for subsequent modeling. These findings demonstrate that the preprocessing approach contributes to the stability and predictive performance of the model.

Table 5 presents the evaluation of the TGA data generation model at different temperatures. The results indicate that the model performs consistently well under varying temperature conditions.

#### 3.1.2. FT-IR Data Generation Model

Table 6 presents a comparison of the FT-IR data generated by the data generation model at various temperatures and the interpolated data obtained by interpolation.

The comparison results generally show high performance. However, the MAE value at 345 °C is relatively higher than at 260 °C. This issue can be addressed through model optimization or by acquiring additional data.

#### 3.1.3. GC-MS Data Generation Model

Table 7 shows the comparison of the GC-MS data generated by the data generation model at various temperatures and the interpolated data obtained by interpolation.

The model demonstrated strong performance across most temperature ranges. However, at 280 °C, the MAE was relatively high (0.00228), while $R^{2}$ and the correlation coefficient dropped significantly to 0.64181 and 0.82605, respectively. The performance degradation at a specific temperature can be addressed by acquiring additional data for that temperature range.

The analysis of the evaluation tables for each data generation model shows that all models exhibit satisfactory performance. This confirms that the proposed data generation models can address data scarcity in situations with limited data and can be effectively utilized as training data for the final GC-MS prediction model.

### 3.2. Evaluate GC-MS Prediction Model

The trained model was used to predict the GC-MS analysis results for lignin depolymerized at various temperatures during the HTL process. Table 8 presents the predicted results.

The GC-MS prediction results in Table 8 demonstrate that the model effectively captures the compositional changes in lignin depolymerization across different temperatures. Other aromatic compounds (0.2984) had the highest predicted mean proportion, followed by glycerol-derived compounds (0.1479), syringyl (0.1384), and guaiacyl (0.1210). At 280 °C, syringyl (0.0585) and guaiacyl (0.1075) sharply declined, while other aromatics increased (0.3308), indicating that the model recognizes the decomposition of major aromatic monomers and the formation of low-molecular-weight compounds.

Variance analysis revealed that syringyl (0.0038) and guaiacyl (0.0027) showed significant fluctuations, while polycyclic aromatics (0.0013), alkanes (0.0018), and cyclic compounds (0.0021) remained relatively stable. The model also predicted a peak in polycyclic aromatics at 345 °C (0.0528), followed by a decline at 390 °C (0.0062), suggesting high-temperature decomposition. Additionally, alkanes increased at 390 °C (0.0883), fatty acids peaked at 345 °C (0.0729), and glycerol-derived compounds peaked at 365 °C (0.2303) before declining (0.1077 at 390 °C), reflecting thermal degradation trends.

Although the model captures general depolymerization patterns, fluctuations at 280 °C and 345 °C indicate potential limitations in learning nonlinear transformations. Further data collection and model refinement are necessary to improve predictive accuracy.

Table 9 shows the evaluation results assessing the consistency between the interpolated data and the reference data. The table presents the group ratios, MAE, $R^{2}$, and correlation coefficients for each temperature condition.

Notably, at 260 °C and 365 °C, the data indicate low MAE values, high correlation coefficients, and robust $R^{2}$ values, thereby confirming the model’s superior predictive performance under these conditions. In contrast, the results at 280 °C and 345 °C reveal comparatively higher MAE, lower $R^{2}$, and weaker correlation coefficients, clearly demonstrating a decline in performance. These findings not only attest to the interpolation method’s effectiveness in preserving the original data characteristics but also highlight the necessity for further validation using empirical data.

## 4. Conclusions

This study investigated the feasibility of an AI-based model for predicting GC-MS analysis results of depolymerized lignin using FT-IR and TGA data. The primary objective was to establish a cost-effective and efficient analytical methodology that could serve as a viable alternative to conventional GC-MS analysis, which is often associated with high costs and complex sample preparation procedures.

The experimental results showed promising predictive performance, particularly at temperatures of 260 °C ($R^{2}=0.6916$, correlation coefficient = 0.89697) and 365 °C ($R^{2}=0.76062$, correlation coefficient = 0.92887), indicating that the model accurately captures subtle variations in lignin composition under these conditions. However, variability in model performance at 280 °C and 345 °C highlights nonlinear characteristics of lignin decomposition that remain incompletely addressed, necessitating further data acquisition and model refinement.

Compared to conventional GC-MS methods, the proposed approach significantly reduces analytical costs and complexity due to lower initial investments and per-sample processing costs. This advantage is particularly beneficial for large-scale lignin characterization and real-time monitoring in industrial processes, such as biomass conversion and polymer development.

Overall, this study provides compelling evidence supporting the viability and potential impact of AI-driven predictive modeling using FT-IR and TGA data in lignin analysis. To further validate and enhance the generalizability of the proposed model, future research will expand datasets to include diverse lignin substrates and refine feature extraction methods.

In addition, systematic experimental validation through controlled laboratory-scale depolymerization experiments will be conducted, comparing predicted GC-MS profiles against actual measurements. Collaboration with organic chemists will specifically target the optimization of sample preparation methods, structural validation of predicted outcomes, and detailed chemical interpretation of observed nonlinearities at specific temperature ranges. These steps are expected to substantially strengthen the practical utility and robustness of the proposed analytical methodology.

## Figures and Tables

**Table 1:** The proposed TGA data generation model architecture.

Configuration		Output Shape
**Feature Extractor (CNN)**		
Conv1D(1, 16, kernel=3, stride=1, padding=1)	→ ReLU	(batch, 16, input_size)
Conv1D(16, 32, kernel=3, stride=1, padding=1)	→ ReLU	(batch, 32, input_size)
Flatten		(batch, 32 × input_size)
**Fully Connected Layers**		
Linear (32 × input_size, 1024)	→ ReLU	(batch, 1024)
Linear (1024, output_size)		(batch, 761)

**Table 2:** The proposed FT-IR data generation model architecture.

Configuration		Output Shape
**Feature Extractor (CNN)**		
Conv1D (1, 16, kernel=3, stride=1, padding=1)	→ ReLU	(batch, 16, input_size)
Conv1D (16, 32, kernel=3, stride=1, padding=1)	→ ReLU	(batch, 32, input_size)
Conv1D (32, 64, kernel=3, stride=1, padding=1)	→ ReLU	(batch, 64, input_size)
Flatten		(batch, 64 × input_size)
**Fully Connected Layers**		
Linear (64 × 1, 1024)	→ ReLU	(batch, 1024)
Dropout (0.3)		(batch, 1024)
Linear (1024, 3476)		(batch, 3476)

**Table 3:** The proposed GC-MS data generation model architecture.

Configuration		Output Shape
**Feature Extractor (CNN)**		
Conv1D (1, 64, kernel=3, padding=1)	→ ReLU	(batch, 64, input_size)
Conv1D (64, 64, kernel=3, padding=1)	→ ReLU	(batch, 64, input_size)
Conv1D (64, 64, kernel=3, padding=1)	→ ReLU	(batch, 64, input_size)
Global Average Pooling		(batch, 64)
**Fully Connected Layers**		
Linear (64, 128)	→ ReLU	(batch, 128)
Linear (128, 10)	→ Softmax	(batch, 10)

**Table 4:** Wasserstein distance between preprocessed and original TGA data.

Temperature	Wasserstein Distance
250 °C	$1.77\times 10^{-3}$
300 °C	$1.406\times 10^{-3}$
350 °C	$1.036\times 10^{-3}$
400 °C	$1.215\times 10^{-3}$

**Table 5:** Evaluation of the TGA data generation model at various temperatures.

Temperature	MAE	$R^{2}$	Correlation
260 °C	$1.0\times 10^{-5}$	0.99846	0.99927
280 °C	$3.0\times 10^{-5}$	0.99677	0.99856
315 °C	$1.0\times 10^{-5}$	0.99936	0.99974
345 °C	$3.0\times 10^{-5}$	0.99695	0.99895
365 °C	$3.0\times 10^{-5}$	0.99775	0.99930
390 °C	$1.0\times 10^{-5}$	0.99985	0.99994

**Table 6:** Evaluation of the FT-IR generation model at various temperatures.

Temperature	MAE	$R^{2}$	Correlation
260 °C	$2.8\times 10^{-4}$	0.99982	0.99991
280 °C	$4.9\times 10^{-4}$	0.99969	0.99986
315 °C	$9.3\times 10^{-4}$	0.99942	0.99973
345 °C	$1.4\times 10^{-3}$	0.99918	0.99961
365 °C	$1.0\times 10^{-3}$	0.99936	0.99968
390 °C	$4.1\times 10^{-4}$	0.99974	0.99987

**Table 7:** Evaluation of the GC-MS generation model at various temperatures.

Temperature	MAE	$R^{2}$	Correlation
260 °C	$1.2\times 10^{-4}$	0.98418	0.99341
280 °C	$2.3\times 10^{-3}$	0.64181	0.82605
315 °C	$1.3\times 10^{-4}$	0.98445	0.99749
345 °C	$1.3\times 10^{-4}$	0.98645	0.99837
365 °C	$4.2\times 10^{-4}$	0.96308	0.98587
390 °C	$6.8\times 10^{-4}$	0.9756	0.99835

**Table 8:** Prediction results of the GC-MS model at various temperatures (values represent relative proportions).

Temperature	Syringyl	Guaiacyl	Poly Aromatics (C10–C21)	Other Aromatics (C6–C20)	Alkanes	Cyclic	Fatty Acids	Alcohol	Glycerol- Derived	Other
260 °C	0.21477	0.17646	0.05685	0.29564	0.01579	0.04039	0.05028	0.01391	0.07410	0.15986
280 °C	0.05852	0.10754	0.02521	0.33085	0.00343	0.07756	0.00424	0.02082	0.19208	0.09062
315 °C	0.16934	0.09127	0.04828	0.28594	0.00831	0.02845	0.00364	0.01301	0.16618	0.07224
345 °C	0.07758	0.06217	0.05289	0.30754	0.01026	0.03029	0.07295	0.01783	0.11612	0.07224
365 °C	0.15026	0.14106	0.01434	0.32278	0.00312	0.00299	0.08885	0.05081	0.23030	0.02578
390 °C	0.14120	0.12963	0.00629	0.26187	0.08833	0.02704	0.01767	0.01606	0.10773	0.09065

**Table 9:** Evaluation of the prediction results of the GC-MS model.

Temperature	MAE	$R^{2}$	Correlation
260 °C	$2.07\times 10^{-3}$	0.6916	0.89697
280 °C	$4.87\times 10^{-2}$	0.34032	0.69668
315 °C	$4.00\times 10^{-3}$	0.39581	0.76097
345 °C	$1.04\times 10^{-2}$	−0.31819	0.40104
365 °C	$2.19\times 10^{-3}$	0.76062	0.92887
390 °C	$9.72\times 10^{-3}$	0.51835	0.72818

## Data Availability

Data are available upon request to the corresponding authors.

## Appendix A

**Table A1:** Compounds identified through GC-MS analysis in lignin samples.

Groups	Derivative	Chemical Formula
Syringyl	Phenol, 2,6-dimethoxy-	C8H10O3
	4-methylsyringol; 3,5-Dimethoxy-4-hydroxytoluene	C9H12O3
	4-Ethylsyringol; 4-Ethyl-2,6-dimethoxyphenol	C10H14O3
	4-Propylsyringol; 2,6-Dimethoxy-4-propylphenol	C11H16O3
	Syringaldehyde; Benzaldehyde, 4-hydroxy-3,5-dimethoxy-	C9H10O4
	(E)-4-Propenylsyringol (E)-2,6-Dimethoxy-4-(prop-1-en-1-yl) phenol	C11H14O3
	4-Acetylsyringol; Acetosyringon; Ethanone, 1-(4-hydroxy-3,5-dimethoxyphenyl)-	C10H12O4
	Syringylacetone	C11H14O4
	Syringyl alcohol; 3,5-Dimethoxy-4-hydroxybenzeneethanol	C10H14O4
	Butylsyringone	C12H16O4
	Acetyl syringic acid, ethyl ester	C13H16O6
	Propiosyringone; 1-Propanone, 1-(4-hydroxy-3,5-dimethoxyphenyl)-	C11H14O4
	Dihydrosyringenin; 3-Syringylpropanol	C11H16O4
Guaiacyl	Guaiacol; Phenol, 2-methoxy-	C7H8O2
	5-Methylguaiacol; m-Creosol; 2-Methoxy-5-methylphenol	C8H10O2
	4-Ethylguaiacol; Phenol, 4-ethyl-2-methoxy-	C9H12O2
	4-Propylguaiacol; Phenol, 2-methoxy-4-propyl-	C10H14O2
	Benzaldehyde, 3-hydroxy-4-methoxy-	C8H8O3
	Allylguaiacol; Eugenol	C10H12O2
	Guaiacylacetone; 2-Propanone, 1-(4-hydroxy-3-methoxyphenyl)-	C10H12O3
	4-(2-Hydroxyethyl)guaiacol; Homovanillyl alcohol	C9H12O3
	3-(4-guaiacyl)propanol; Benzenepropanol, 4-hydroxy-3-methoxy-	C10H14O3
Poly aromatics	Naphthalene	C10H8
	7-Methoxy-1-naphthol	C11H10O2
	2-Naphthalenol, 3-methoxy-	C11H10O2
	1,6-Dimethoxynaphthalene	C12H12O2
	Naphthalene, 2,3-dimethoxy-	C12H12O2
	1,6-Dimethoxynaphthalene	C12H12O2
	Retene	C18H18
	2-Isopropyl-10-methylphenanthrene	C18H18
	Methyl dehydroabietate	C21H30O2
	8-Isopropyl-1,3-dimethylphenanthrene	C19H20
Other aromatics	Phenol	C6H6O
	p-Cresol	C7H8O
	o-Cresol; Phenol, 2-methyl-	C7H8O
	Creosol	C8H10O2
	Catechol	C6H6O2
	1-Propanone, 1-(5-methyl-2-thienyl)-	C8H10OS
	2-Acetyl-4-methylphenol; o-Acetyl-p-cresol	C9H10O2
	3-methoxycatechol; 1,2-Benzenediol, 3-methoxy-	C7H8O3
	Hydroquinone	C6H6O2
	4-Methylcatechol; 1,2-Benzenediol, 4-methyl-	C7H8O2
	3-Methylcatechol; 1,2-Benzenediol, 3-methyl-	C7H8O2
	Phenol, 4-methoxy-3-methyl-	C8H10O2
	2,3-Dimethoxyphenol	C8H10O3
	Phenol, 3,4-dimethoxy-	C8H10O3
	5-Methoxy-m-cresol; 3-Methoxy-5-methylphenol	C8H10O2
	2,6-Dimethoxyhydroquinone	C8H10O4
	1,4-Benzenedicarboxaldehyde, 2-methyl- 2-Methylterephthalaldehyde	C9H8O2
	Ethanone, 1-(2-hydroxy-5-methylphenyl)-	C9H10O2
	Ethanone, 1-(2-hydroxy-6-methoxyphenyl)-	C9H10O3
	1,2,3-Trimethoxybenzene	C9H12O3
	4-Ethylcatechol	C8H10O2
	1,4-Benzenediol, 2,3,5-trimethyl- Trimethylhydroquinone	C9H12O2
	Ethanone, 1-(2,3,4-trihydroxyphenyl)-	C8H8O4
	Vanillin	C8H8O3
	3-Ethoxy-4-methoxyphenol	C9H12O3
	Phenol, 2-methoxy-4-(2-propenyl)-, acetate; Aceto eugenol	C12H14O3
	3-Acetylphenol; Ethanone, 1-(3-hydroxyphenyl)-	C8H8O2
	2-methoxy-5-acetylphenol; Ethanone, 1-(3-hydroxy-4-methoxyphenyl)-	C9H10O3
	Apocynin	C9H10O3
	Benzene, 1,2,3-trimethoxy-5-methyl-	C10H14O3
	2-Propanone, 1-(4-hydroxy-3-methoxyphenyl)-	C10H12O3
	3-Hydroxy-4-methoxybenzoic acid	C8H8O4
	Flopropione	C9H10O4
	3,4-Dimethoxyphenylacetone	C11H14O3
	1-Propanone, 1-(4-hydroxy-3-methoxyphenyl)-	C10H12O3
	Butyrovanillone	C11H14O3
	Homovanillic acid	C9H10O4
	Benzenepropanol, 4-hydroxy-3-methoxy-	C10H14O3
	Phenol, 2-methoxy-4-methyl-6-[propenyl]-	C11H14O2
	2,3-Dimethoxy-5-aminocinnamonitrile	C11H12N2O2
	5-(3-Hydroxypropyl)-2,3-dimethoxyphenol	C11H16O4
	Asarone	C12H16O3
	Benzene, 1,2,3-trimethoxy-5-(2-propenyl)-	C12H16O3
	3,4-Divanillyltetrahydrofuran	C20H24O5
	1-(2,4-Dihydroxyphenyl)-2-(3,4-dimethoxyphenyl)ethan one	C16H16O5
	1-(2,4-Dihydroxyphenyl)-2-(3,5-	C17H18O5
	Dehydroabietate	C20H28O2
	3,4-Dimethoxyphenol, 2- methylpropionate	-
Alkanes (Paraffins)	Propane, 1,1-diethoxy-	C7H16O2
	1,3,5-Trioxane	C3H6O3
	Propanal ethyl isopentyl acetal 1-(1-Ethoxypropoxy)-3-methylbutane	C10H22O2
Cyclic	Oxazolidin-2-one	C3H5NO2
	Butyrolactone	C4H6O2
	2-Cyclopenten-1-one, 3-methyl-	C6H8O
	1,2-Cyclopentanedione, 3-methyl-	C6H8O2
	2-Cyclopenten-1-one, 2-hydroxy-3-methyl-	C6H8O2
	2-Cyclopenten-1-one, 2,3-dimethyl-	C7H10O
Fatty Acids	Propanoic acid	C3H6O2
	Butanoic acid, 4-hydroxy-	C4H8O3
	Methyltartronic acid	C4H6O5
	Lactic acid; Propanoic acid, 2-hydroxy-, ethyl ester	C5H10O3
	Pentanoic acid, 4-oxo-	C5H8O3
	Pentanoic acid, 4-oxo-, ethyl ester	C7H12O3
	Butanoic acid, anhydride	C8H14O3
	Butanoic acid, 2-methylpropyl ester	C8H16O2
	Propanoic acid, 2-methyl-, anhydride	C8H14O3
	Pentanoic acid, 4-oxo-, 2-methylpropyl ester	C9H16O3
	Dodecanoic acid, methyl ester Pentanoic acid, 2-methyl-4-oxo-	C13H26O2
Alcohols	1,3-Propanediol	C3H8O2
	Ethanol, 2,2’-oxybis-	C4H10O3
	1,2-Propanediol, 3-methoxy-	C4H10O3
	1-Propanol, 2-(2-hydroxypropoxy)-	C6H14O3
Glycerol-derived	3-Ethoxy-1,2-propanediol; Glycerol 1-ethyl ether	C5H12O3
	Glycerol triethyl ether	C9H20O3
	1,3-Dioxolane-4-methanol, 2-ethyl-	C6H12O3
	Glycerin	C3H8O3
	1,2,3-Propanetriol, 1-acetate	C5H10O4
	Glycerol 1,2-diacetate	C7H12O5
	Alpha-monopropionin	C6H12O4
	Hydroxyacetone; 2-Propanone, 1-hydroxy-	C3H6O2
	Ethylene glycol Formate Isobutyrate	C7H12O4
	2,3-dihydroxypropyl isobutyrate	C7H14O4
